# An artificial intelligence model (euploid prediction algorithm) can predict embryo ploidy status based on time-lapse data

**DOI:** 10.1186/s12958-021-00864-4

**Published:** 2021-12-13

**Authors:** Bo Huang, Wei Tan, Zhou Li, Lei Jin

**Affiliations:** Reproductive Medicine Center, Tongji Hospital, Tongji Medicine College, Huazhong University of Science and Technology, 430030 Wuhan, People’s Republic of China

**Keywords:** AI, Ploidy status, time-lapse, PGT, Prediction

## Abstract

**Background:**

For the association between time-lapse technology (TLT) and embryo ploidy status, there has not yet been fully understood. TLT has the characteristics of large amount of data and non-invasiveness. If we want to accurately predict embryo ploidy status from TLT, artificial intelligence (AI) technology is a good choice. However, the current work of AI in this field needs to be strengthened.

**Methods:**

A total of 469 preimplantation genetic testing (PGT) cycles and 1803 blastocysts from April 2018 to November 2019 were included in the study. All embryo images are captured during 5 or 6 days after fertilization before biopsy by time-lapse microscope system. All euploid embryos or aneuploid embryos are used as data sets. The data set is divided into training set, validation set and test set. The training set is mainly used for model training, the validation set is mainly used to adjust the hyperparameters of the model and the preliminary evaluation of the model, and the test set is used to evaluate the generalization ability of the model. For better verification, we used data other than the training data for external verification. A total of 155 PGT cycles from December 2019 to December 2020 and 523 blastocysts were included in the verification process.

**Results:**

The euploid prediction algorithm (EPA) was able to predict euploid on the testing dataset with an area under curve (AUC) of 0.80.

**Conclusions:**

The TLT incubator has gradually become the choice of reproductive centers. Our AI model named EPA that can predict embryo ploidy well based on TLT data. We hope that this system can serve all in vitro fertilization and embryo transfer (IVF-ET) patients in the future, allowing embryologists to have more non-invasive aids when selecting the best embryo to transfer.

**Supplementary Information:**

The online version contains supplementary material available at 10.1186/s12958-021-00864-4.

## Introduction

In the field of assisted reproductive technology (ART), selecting embryos with the highest developmental potential has always been a research hotspot, and it is also the direction of all embryologists’ efforts [1]. There are many methods for selecting embryos that have been reported, and these methods can be roughly divided into non-invasive and invasive.

Non-invasive methods include proteomics and metabolomics research, as well as developmental dynamics research [2–5]. Compared with invasive methods, non-invasive embryo selection methods are undoubtedly more natural and safer. Some researchers have questioned the safety of PGT’s invasive biopsy method and its impact on embryo implantation potential, because there are reports that invasive removal of cells from preimplantation embryos may interfere with embryo development [6, 7]. In addition, invasive biopsy requires special equipment and well-trained embryologists, which are different from routine in terms of time and cost. In short, although invasive biopsy is still the cornerstone of PGT, there are non-invasive methods to help select embryos when invasive biopsy is unnecessary. We believe that no one will be willing to try invasive methods.

Among non-invasive methods, time-lapse technology (TLT) can already provide us with a lot of information about embryonic development dynamics [8]. It allows embryologists to progress from the previous static evaluation to the dynamic evaluation, and provides a great help for embryo selection [9]. To this end, European Society of Human Reproduction and Embryology (ESHRE) Time-lapse working group also gave a systematic introduction and evaluation of TLT [10, 11].

Invasive methods mainly refer to PGT technology. PGT for aneuploidy (PGT-A) can provide embryo ploidy information, which is very important for embryo implantation. However, PGT is not available in all countries and there remains some controversy regarding its cost-effectiveness or clinical necessity [12–15]. As mentioned, TLT provides information on all the kinetic parameters of embryonic development. If we can find ploidy-related indicators from the TLT data, it will be very beneficial for all patients with routine in vitro fertilization and embryo transfer (IVF-ET). In other words, the ability to know or predict ploidy information with a high probability without invasive biopsy can greatly improve the efficiency of embryo selection and further improve clinical outcomes. There have been comprehensive review articles discussing the relationship between TLT and ploidy [16, 17]. The conclusion shows that although the relevant parameters have been reported, the desired results have not been achieved, and more research is still needed.

For massive amounts of data, artificial intelligence (AI) can help us, especially in the analysis and classification of embryonic developmental dynamics parameters [18]. In terms of clinical pregnancy research, articles on AI applications have been reported. Researchers use deep learning through embryo pictures and kinetic parameters to achieve good prediction results (area under curve, AUC>0.9) [19, 20]. However, there are not many reports on the research of artificial intelligence in ploidy, which is also the goal that various research teams strive to achieve. Recently, Chavez-Badiola et al.[21] and Bori et al. [22] reported the use of AI and TLT to predict embryo ploidy information. These are very important research results in this field. As stated in the guide: ‘There is little doubt that the future of AI and TLT will incorporate some degree of machine learning, to facilitate complex analysis of large data sets, which will likely reveal currently unidentified combinations of visual markers.’ [10]. More research needs to be reported to improve the efficiency and accuracy of ploidy prediction.

The aim of this study is to develop a model named euploid prediction algorithm (EPA) that can predict embryo ploidy through TLT data, and provide more help for embryo selection or sorting in conventional IVF-ET cycles.

## Materials and methods

### Study design and participants

**Fig. 1:**
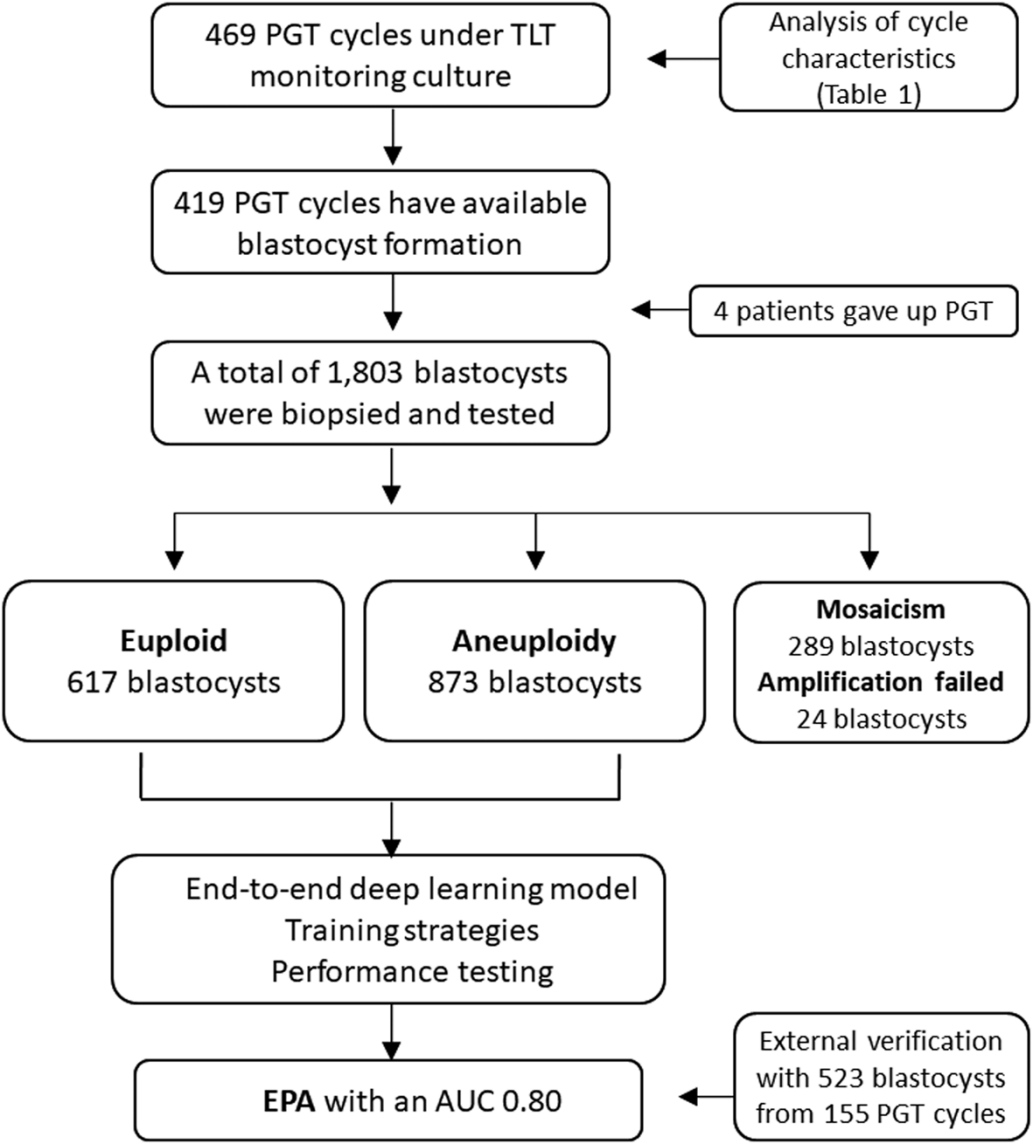
Flow chart of the study design

In this single-center cohort study, a total of 469 PGT cycles and 1803 blastocysts were included in the study. The time-lapse embryo data used in this study are collected from Reproductive Medicine Center of Tongji Hospital, Huazhong University of Science and Technology, Wuhan, China. All embryo images are captured during 5 or 6 days after fertilization before biopsy or cryopreservation by Embryoscope Plus time-lapse microscope system (Vitrolife, Denmark) from April 2018 to November 2019. All patients signed written informed consent and underwent the routine clinical treatment performed in our center. No additional intervention was performed. The study conformed to the Declaration of Helsinki for Medical Research involving Human Subjects. It was approved by the Ethical Committee of Reproductive Medicine Center, Tongji Hospital, Tongji Medicine College, Huazhong University of Science and Technology. The design and process of the study are shown in Fig. 1.

### ICSI and embryo incubation

The procedures for intracytoplasmic sperm injection (ICSI) have been described previously [23]. Briefly, during the ICSI processing, cumulus cells and the corona radiata of the oocytes were removed by brief exposure to hyaluronidase 2-3 h after retrieval (HYASE, Vitrolife); ICSI was performed on metaphase II oocytes as observed under an inverted microscope. All injected eggs were placed into timelapse incubator immediately (Embryoscope Plus). Then, the fertilized oocytes were continuously cultured at 5%CO_2_, 5%O_2_ and 37ºC for 2 more days (G1-plus, Vitrolife, Sweden). The culture conditions of the embryos included in this study were the same. All of the embryos were checked on the morning of day 3 after oocyte retrieval. Then the embryos were changed to blastocyst medium to continue culture until Day5/6 (G2-plus, Vitrolife, Sweden). The blastocyst biopsy is done on the Day5/6. A small hole is made in the zona pellucida using a laser just prior to biopsy, and then a mechanical cutting method is used to obtain 3-6 trophectoderm cells.

### NGS analysis

Details of next-generation sequencing technology (NGS) analysis procedure have been described previously [24]. Briefly, a multiple annealing and looping-based amplification cycles (MALBACs) based single-cell whole-genome amplification (WGA) protocol was used to amplify the samples following the commercial kit protocol from *Yikon Genomics*. A series of DNA fragmentation, amplification, tagging, and purification were completed. Then, the products were purified. The final library was sequenced using Life Technologies Ion Proton platform at approximately 0.04×genome depth. This sequencing throughput yields reproducible copy number variation (CNV) with approximately 4 MB resolution to detect variation. The threshold for aneuploidy detection was set to be greater than 70%. The threshold for mosaic detection varies from chromosomes. For chromosomes 13, 16, 18, and 21, the lower limit was 30%, for the 19 chromosome, lower limit was 50%, for others, lower limit was 40%. The value below the lower limit indicates a euploidy.

### Dataset

All the euploid or aneuploid embryo are used as data sets. We merge embryo images, embryo development information, and patient clinical information to form a complete data set. The kinetic parameters of each blastocyst are manually marked by Dr. Huang. All the marked data in this study were reviewed by Dr. Tan. The data set is divided into training set, validation set and test set. The training set is mainly used for model training, the validation set is mainly used to adjust the hyperparameters of the model and the preliminary evaluation of the model, and the test set is used to evaluate the generalization ability of the model.

**Fig. 2:**
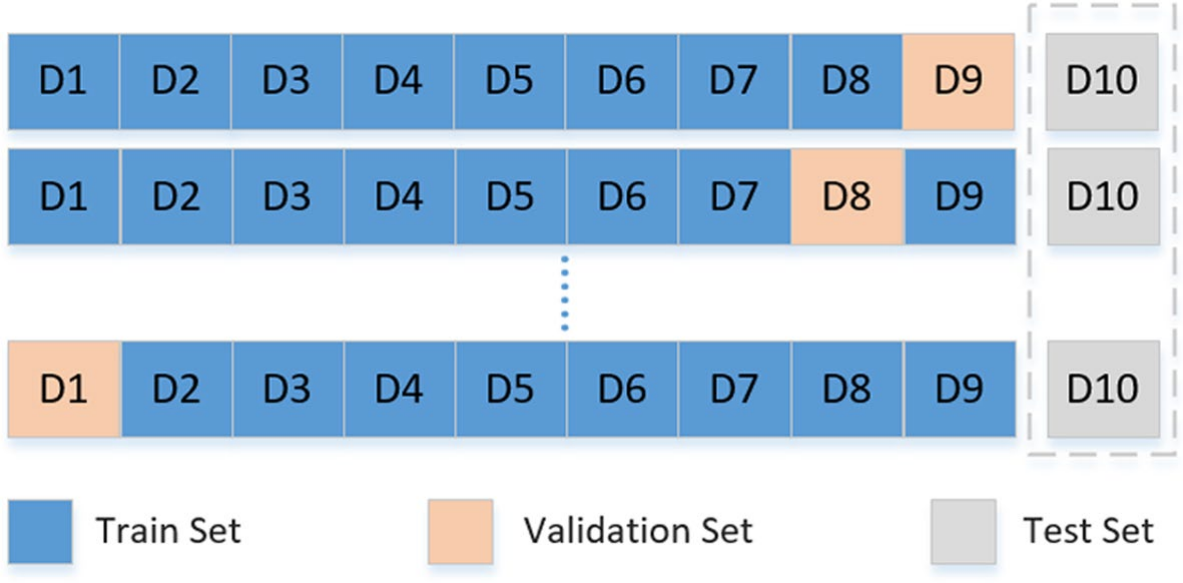
The distribution of the data set. the all data set is divided into ten parts (D1-D10). Among these parts, D10 is always reserved as a test set to improve the generalization ability of the test model. The rest is used for training. The D9, D8, … D1 take turns to be used as the verification set in order to evaluate models

In order to reduce the risk of over-fitting, we adopt the k-fold cross-validation method to ensure that each sample has only one chance to be included in the training set or test set during each iteration. In this study, k is set to 10 based on empirical values. In other words, the all data set is divided into ten parts (D1-D10). Among these parts, D10 is always reserved as a test set to improve the generalization ability of the test model. The rest is used for training. The D9, D8, … D1 take turns to be used as the verification set in order to evaluate models. (Figure 2). For better verification, we used data other than the training data for external verification. We used the PGT cycle data from December 2019 to December 2020. A total of 155 PGT cycles and 523 blastocysts were included in the verification process.

### Algorithm Description

**Fig. 3:**
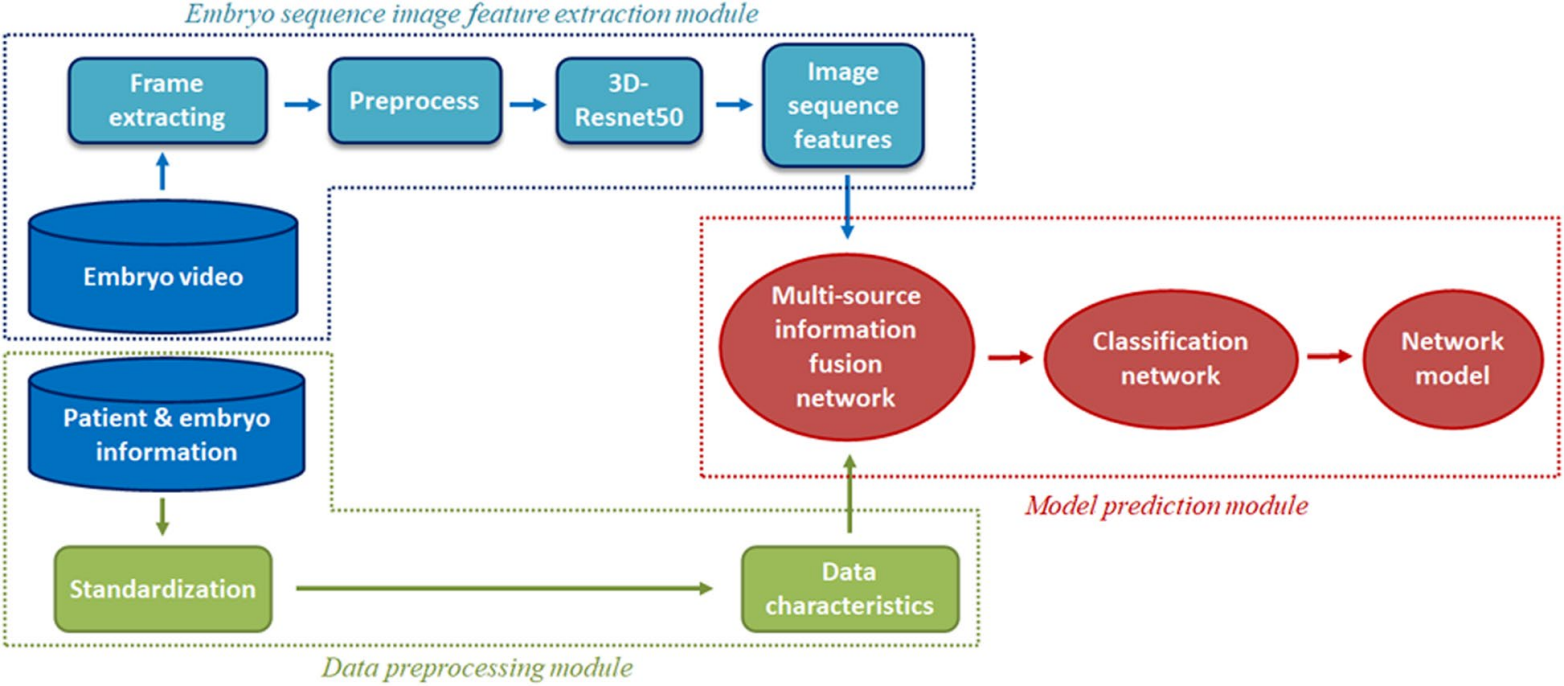
The brief schematic diagram of algorithm

This algorithm is mainly divided into three modules. The first module is mainly used to extract embryo sequence image features (a), the second module is mainly used to normalize embryo data and clinical data features (b), and the third module is mainly used for feature fusion and model prediction (c). The brief schematic diagram is shown in Fig. 3.

#### Embryo sequence image feature extraction module

The embryo images taken in the TL incubator are sequentially input into the network model along with the embryonic development process. Before the model training calculation, in order to speed up the convergence speed of the model and improve the accuracy of the model, it is necessary to perform preliminary processing operations on the image, including the embryo image is incomplete and the embryo image is blurred. According to the consensus of embryology experts and the embryo development time reported in the literature [10, 25], this article intercepted embryo images of 26-28 h, 40-45 h, 55-65 h, 85-95 h, 110-116 h and 132.5-136 h.

According to the embryo development video, 64 non-adjacent images are randomly selected with a step size of 2 to contain as much as possible the images of the embryo at different stages. The 64 embryo images were adjusted to a resolution of 112 × 112, and the image values were normalized. This research uses the first 49 layers of 3D convolutional network of 3D-resnet50 [26] as the video sequence feature extraction network. Input the preprocessed image into the network, after going through a 7 × 7 × 7 three-dimensional convolution network, and then passing through 3, 4, and 6 three-dimensional convolution blocks in turn, the 2048-dimensional features of sequence image is obtained after adaptive pooling.

#### Data preprocessing module

The kinetic parameters during embryo development are input as embryo data. At the same time, the patient’s age is input as clinical data. In order to reduce the influence of embryonic data and clinical data’s numerical value prediction model parameters, this paper uses formula (1) to standardize the data.

1 $${x}^{\text{'}}=\frac{x-\stackrel{-}{x}}{\delta }$$

$\stackrel{-}{x}$represents the average value of the variable, and δ represents the variance of the variable. Replace the missing items with 0.

#### Model prediction module

In order to better integrate multiple feature information, this study proposes to use a fully connected layer network as a multi-source information fusion network. The 2048-dimensional video sequence features extracted by 3D-resnet50 are spliced with the standardized clinical and embryo data features to form a new feature vector, which is processed by the multi-source information fusion network to obtain the fused feature vector, and then input to the second layer predictive classification of models in a fully connected network. Select the label corresponding to the maximum value of the two output result values as the final output value of the embryo.

### AUC

The AUC ranges from 0.5 to 1.0, representing the predictive power of the binary classifier. An AUC of 0.5 means completely random selection, and an AUC of 1 means complete discrimination. The higher the AUC, the more favorable the trade-off between sensitivity and specificity [19]. The quantitative value of AUC can also be simply interpreted as the probability that the binary classifier will score randomly selected euploid embryos higher than that of randomly selected aneuploid embryos. Therefore, AUC is the most suitable benchmark for the ability of a binary classifier to rank embryos based on the likelihood of embryo ploidy status.

## Results

From April 2018 to November 2019, a total of 469 PGT cycles used the time-lapse culture system. Among them, 419 cycles have available blastocyst. In 4 cycles, the patient gave up PGT. The clinical characteristics of PGT cycles were shown in Table 1. In the end, a total of 415 cycles and 1803 blastocysts were included in the study. 1,803 blastocysts were biopsied and sequenced. 1,779 blastocysts had test results, and the detection rate was 99%. The results showed that 617 blastocysts are euploid, 873 blastocysts are aneuploid, 289 blastocysts are mosaic. We only select euploid and aneuploidy samples for this study.

**Table 1:** Clinical characteristics of PGT cycles

Parameter	
No. of cycles/patients	469
No. of cycles with available blastocysts (%)	419 (89.3)
No. of cycles cancelled (%)	4 (0.8)
No. of cycles included in the study	415
Age (y)	30.8 ± 4.5
Duration of infertility (y)	2.6 ± 2.5
Basic FSH	7.3 ± 2.5
Basic AMH	5.2 ± 3.7
BMI	22.2 ± 3.1
Duration of stimulation (days)	10.3 ± 1.8
No. of oocytes	6,217
No. of matured oocytes	5,026
No. of two pronucleus (2pn)	3850
Fertilization rate (%)	76.6
No. of available blastocyst	1,803
No. of available blastocysts per cycle	4.34
Euploid (%)	617 (34.2)
Aneuploidy (%)	873 (48.4)
Mosaicism (%)	289 (16.0)
Amplification failed (%)	24 (1.3)

**Fig. 4:**
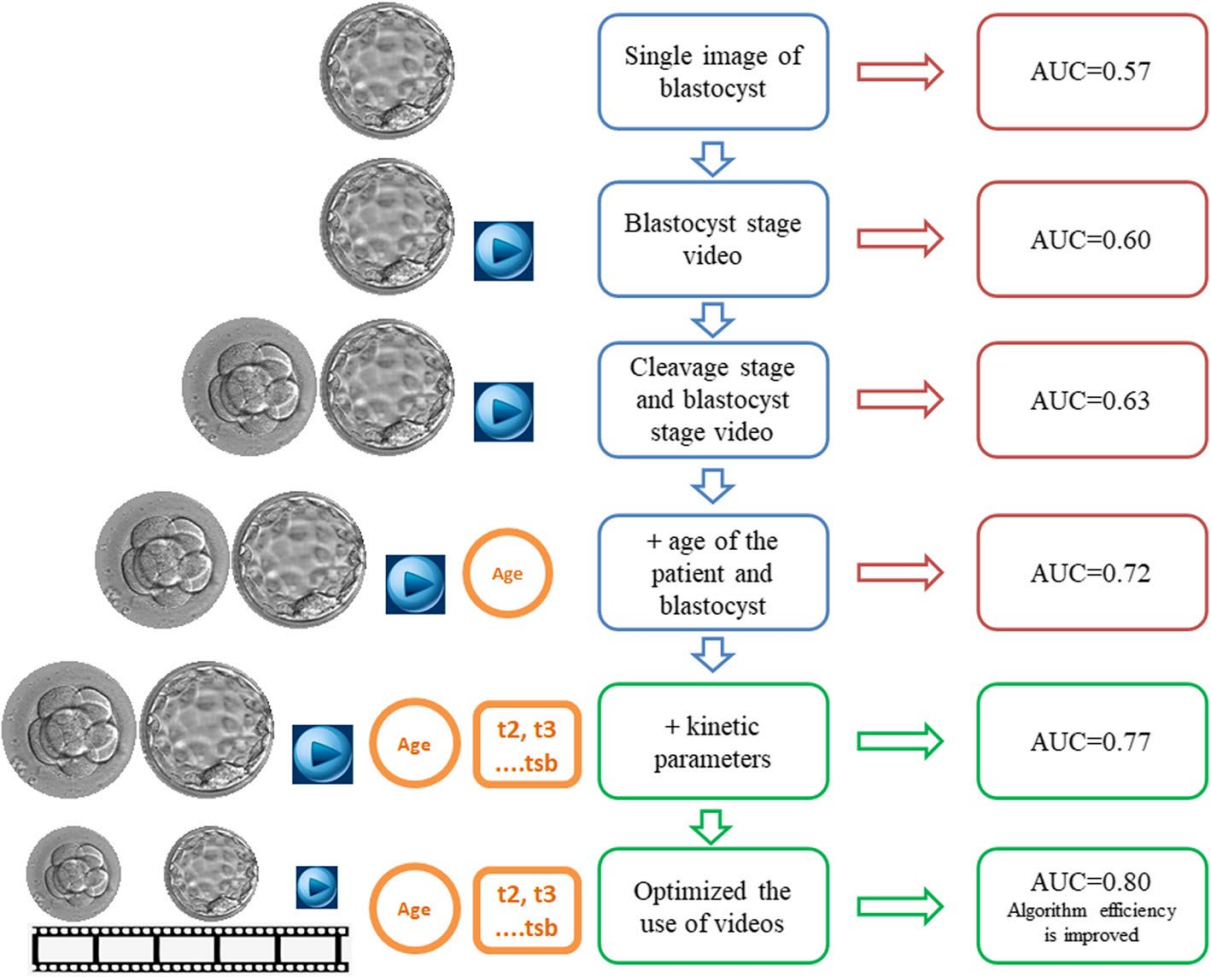
Schematic diagram of Euploid Prediction Algorithm (EPA) research process

**Fig. 5:**
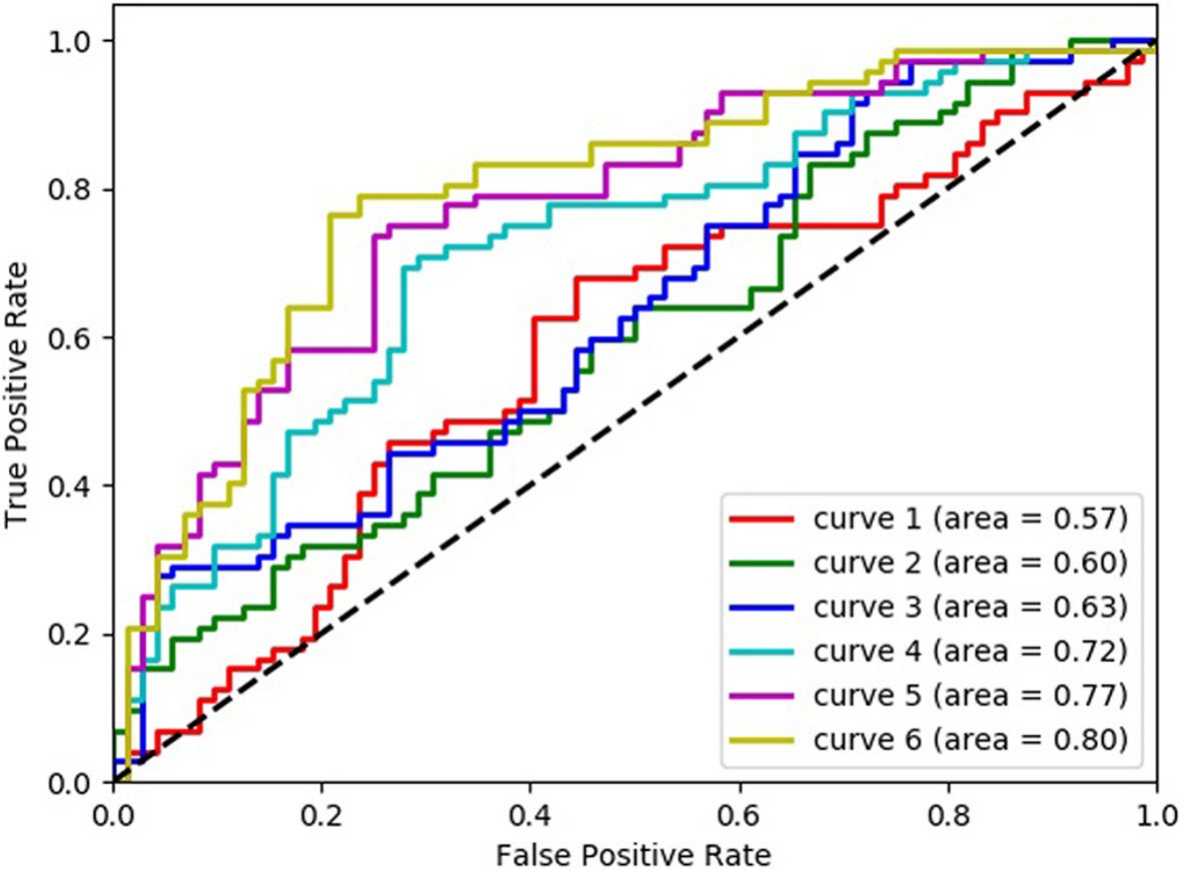
Algorithm’s performance: receiver operating characteristic (ROC) curve. Curve 1, using a single picture before transfer at the blastocyst stage. Curve 2, using blastocyst stage video file. Curve 3, using entire video files of the cleavage stage and the blastocyst stage. Curve 4, adding the age data based on curve 3. Curve 5, adding kinetic data of embryo based on curve 4. Curve 6, optimizing the use of videos based on curve 5

As mentioned in Fig. 4, the development of EPA research tried a variety of data set test models. Initially using the last single picture before biopsy at the blastocyst stage, the tested AUC was 0.57. Then we used the video of the blastocyst stage, which is the video of the embryo at 70 h until before biopsy, and the tested AUC was 0.60. Then we used entire video files of the cleavage stage and the blastocyst stage, and the tested AUC increased to 0.63. Adding the age of the patient and the age of blastocyst (Day5 or Day 6), the AUC can be increased to 0.72. In addition, we add kinetic parameters, the resulting AUC of EPA to predict euploid on the testing dataset was 0.77. Finally, in order to improve the efficiency of the algorithm, we further optimized the use of videos and only use videos from a few time periods, the resulting AUC of EPA to predict euploid on the testing dataset was 0.80. These results were shown in Fig. 5. An example of the predictive calculation process was in the Supplementary Data. Among the 155 PGT cycles used for external verification, 21 of them did not undergo a biopsy because there was no blastocyst formation, so the final number of used for external verification was 134 cycles. These cycles have formed a total of 523 blastocysts. They were all biopsied and sequenced. Among them, 6 blastocysts failed to be amplified. In the end, 517 blastocysts had results, 246 were euploid, 221 were aneuploid, and 50 were mosaic. We use the data of euploid embryos and aneuploid embryos to verify the algorithm. The confusion matrix to represent predictions made by EPA for ploidy status in the test data set was shown in Table 2.

**Table 2:** The confusion matrix to represent predictions made by Euploid Prediction Algorithm (EPA) for ploidy status in the test data set

	True Euploid	True Aneuploid
EPA tested Euploid	193	46
EPA tested Aneuploid	53	175
Total tested embryos	246	221

## Discussion

Artificial intelligence (AI) can help humans in a variety of clinical applications, because it has the ability to quickly learn from large data sets (such as medical images), and because it can weigh variables (high-precision variable weights) and improve in clinical practice medical efficiency and accuracy [27, 28]. In recent years, the research of AI in the field of assisted reproduction is very hot. This can be seen from the number of submissions of annual proceedings of the American Society of Reproduction and European Society of Human Reproduction and Embryology [13]. Embryologists in each laboratory have their own evaluation system and personal experience on how to select embryos with the most developmental potential. But for the increasing amount of embryonic development data from TLT, it would be better if AI can contribute to it.

For embryo ploidy, the assistance of AI is another key research direction. We know that PGT technology can allow embryologists to know the ploidy of embryos before transfer, and better help patients with repeated implantation failure (RIF), recurrent abortion and advanced age. Wouldn’t it be better if this technology could serve all IVF patients? However, biopsy is an invasive procedure for embryos. Therefore, it will be very important if there is a technology that can replace the biopsy technology to know the ploidy of the embryo. Each research team is working hard for this. The AI model (ERICA) reported by Chavez-Badiola et al. can help embryologists successfully sort blastocysts based on their predicted ploidy during embryo selection [21]. The accuracy of the prediction is 0.70, among which the positive predictive value is 0.79 for predicting euploidy. This result is very good in the study of euploid prediction. They used a total of 1231 images of embryos, which were derived from a single image taken by two inverted microscopes of different models on day 5/6. Due to image quality, 946 images (77%) were finally used. As for the source of the images, this study is different from them. In this study, the embryo images we used are all from the same model of TL incubator, the pixels of the images are the same, and there is a complete embryo development process video. It allows us to use different embryonic development parameters or choose different development time periods to optimize and improve the algorithm when building the AI model.

Before using AI to find the prediction method of embryo ploidy state, many research teams have been paying attention to the relationship between embryo development kinetic parameters and ploidy state. Reignier et al. [16] made a systematic review of these studies. A total of 13 studies were included in the analysis. Ten of the studies proved that the ploidy state of the embryo is related to the dynamics of embryonic development, and the other three studies did not find a significant correlation. In general, morphokinetic parameters can help distinguish between euploid and aneuploid embryos, but they are not sufficient to replace PGT. It can only be said that the morphokinetic assessment together with PGT may ultimately help identify euploid embryos with the highest in vitro development potential. This review also gives us other thoughts. If we can comprehensively consider information such as kinetic parameters, patient age, embryo age, and ploidy status, there may be more accurate or more practical methods to help us select embryos that are more likely to be euploid in vitro.

At the very beginning of our AI model building, we also tried to use the last blastocyst image to build the model. Without adding embryo and clinical information, our algorithm only achieved AUC of 0.57. We used the complete embryo development video (cleavage stage and blastocyst stage), AUC can be increased to 0.63. Then we join the age of the patient and the age of blastocyst, the AUC can be increased to 0.72. Considering that there are reports in the literature that kinetic parameters are related to ploidy, such as t2 (time of cleavage to 2-cell), t3 (time of cleavage to 3-cell), t5 (time of cleavage to 5-cell), cc2 (duration time of the second cell cycle), cc3 (duration time of the third cell cycle), s2 (synchronicity of the two blastomere divisions within the second cell cycle), tM (time of the formation of morula), tSB (time of Initiation of blastulation), tB (time of blastocyst), tEB (time of expanded blastocyst) [29–37]. We tried to add kinetic parameters to improve the predicted outcome. After repeated experiments, the resulting AUC of EPA to predict euploid on the testing dataset was 0.77. However, in this case, there will be a problem, that is, the entire prediction model process is relatively time-consuming, because the use of the entire video of embryonic development will increase the amount of calculation, and the calculation of 4 blastocysts takes almost 180 s. Therefore, we further optimized the use of videos and only use videos from a few time periods, so that the predicted AUC can reach 0.80 and the calculation time can be reduced to 70 s for 4 blastocysts. This speed is a bit slower than Chavez-Badiola’s research [21], which may be related to the difference in algorithms and source data. However, we believe that with the continued optimization of the algorithm, this calculation speed will be further improved.

In general, for algorithm verification, 10% of the training set data is used for verification [19, 21]. We believe that this is internal verification to some extent, but we still need external verification. External verification is usually one of the contents not carried out in most of the current research. Therefore, as one of the highlights of this research, we specifically added data other than training samples to verify the performance of the EPA. We conducted verification experiments on the data of 155 PGT cycles, 246 euploids and 221 aneuploidies in our center from December 2019 to December 2020, and the results showed that EPA’s euploid prediction accuracy (78.5%) It has been well verified (Table 2). To the best of our knowledge, this study is currently the report with the largest sample size and the best prediction accuracy. In the future, we will design a randomized controlled experiment to further verify the accuracy and practicability of EPA.

Although this study obtained a high predicted AUC, it still has several limitations. First of all, it is mainly reflected in the algorithm process, we have added manually annotated kinetic parameters. There are subjective differences in manual annotation of kinetic parameters[38], which requires laboratory embryologists to be very experienced and able to unify embryo judgment standards. At the same time, this may cause inconvenience to the rapid operation of the entire algorithm and future clinical applications. Secondly, although the sample size of the study is the largest observed (to date), AI algorithms might be trained on a larger database to avoid overfitting. This requires us to accumulate data, or to carry out multi-center data algorithm development research. Another limitation is that our data source is all embryo data of PGT patients. Whether the prediction system derived from this data source can be applied to other infertility patients. This requires longer time and well-designed experiments to verify.

## Conclusions

At present, the TL incubator has gradually become the choice of reproductive centers when they need to update their equipment [39]. We believe that there will be more and more AI research data from TL, so that the results of different laboratories can have a better parallel comparison. Our AI model (EPA) has added clinical and embryonic TL data to make this system more interpretable. It is essentially different from the ‘black box’[40] AI model that usually only has input and output. Our follow-up work will continue to increase data samples to further improve the prediction level, while reducing image usage to optimize calculation time. However, whether this algorithm can calculate data obtained from other TLT devices requires more research. In addition, our data source is all embryo data of PGT patients. Whether the prediction system derived from this data source can be applied to other infertility patients. This also requires longer time and well-designed experiments to verify. In the future, we hope that this system can serve all IVF-ET patients, allowing embryologists to have more non-invasive aids when selecting the best embryo to transfer.

## Supplementary Information

**Additional file 1.**

**Additional file 2.**

## Data Availability

The datasets used and/or analysed during the current study are available from the corresponding author on reasonable request.

## Abbreviations

TLT: time-lapse technology
AI: artificial intelligence
PGT: preimplantation genetic testing
PGT-A: preimplantation genetic testing for aneuploidy
EPA: euploid prediction algorithm
AUC: area under curve
IVF-ET: in vitro fertilization and embryo transfer
ICSI: intracytoplasmic sperm injection
NGS: next-generation sequencing technology
MALBACs: multiple annealing and looping-based amplification cycles
WGA: whole-genome amplification
CNV: copy number variation
