# Supplementary figures and images for: An artificial intelligence model (euploid prediction algorithm) can predict embryo ploidy status based on time-lapse data
Source: Reprod Biol Endocrinol. 2021 Dec 13;19:185. doi: 10.1186/s12958-021-00864-4 (PMC8667440; doi:10.1186/s12958-021-00864-4)

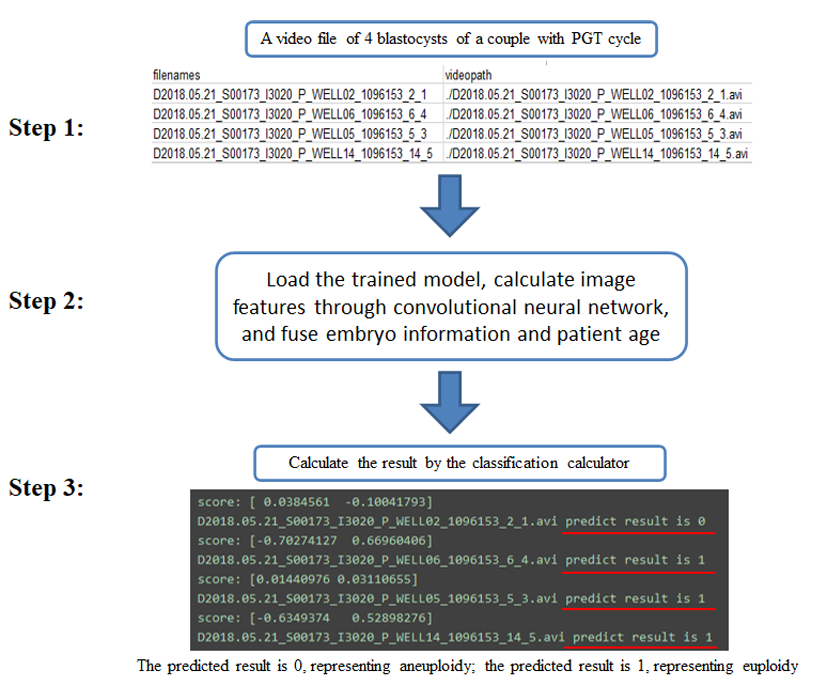

Supplement: Supplementary file 2 — Additional file 2. [file 12958_2021_864_MOESM2_ESM.tif]
